# Crystal structure of 1,3-di­cyclo­hexyl-1-[3-(pyren-1-yl)prop­anoyl]urea

**DOI:** 10.1107/S2056989015015996

**Published:** 2015-09-12

**Authors:** Edgar González-Juárez, Marisol Güizado-Rodríguez, Victor Barba, Hugo Tlahuext

**Affiliations:** a Centro de Investigación en Ingeniería y Ciencias Aplicadas, Universidad Autónoma del Estado de Morelos, Av. Universidad No. 1001 Col. Chamilpa, CP 62209, Cuernavaca Mor., Mexico; b Centro de Investigaciónes Químicas, Universidad Autónoma del Estado de Morelos, Av. Universidad No. 1001 Col. Chamilpa, CP 62209, Cuernavaca Mor., Mexico

**Keywords:** crystal structure, *N*,*N*′-di­cyclo­hexyl­carbodimide, *N*,*N*′-di­cyclo­hexyl­urea, hydrogen bonds

## Abstract

In the title compound, C_33_H_38_N_2_O_2_, each of the cyclo­hexyl rings adopts a chair conformation. The two planes involving carbonyl groups, C—(C=O)—N and N—(C=O)—N, are oriented at a dihedral angle of 62.28 (10)°. In the crystal, two neighboring mol­ecules are linked by a pair of N—H⋯O inter­actions, generating an inversion dimer. The dimers are inter­connected by C—H⋯O hydrogen bonds into a supra­molecular chain along the *a-*axis direction.

## Related literature   

For the synthesis of the title compound, see: Abd-El-Aziz *et al.* (2013 ▸). For the syntheses of *N*,*N*′-di­cyclo­hexyl­carbodi­imide and *N*-acyl-*N*,*N*′-di­cyclo­hexyl­urea, see: Zhu *et al.* (2008 ▸); Gonçalves & Balogh (2006 ▸); Kaiser *et al.* (2008 ▸); Slebioda (1995 ▸). For related crystal structures, see: Chérioux *et al.* (2002 ▸); Cai *et al.* (2009 ▸); Imhof (2007 ▸); Dhinaa *et al.* (2010 ▸); Pinheiro *et al.* (2011 ▸).
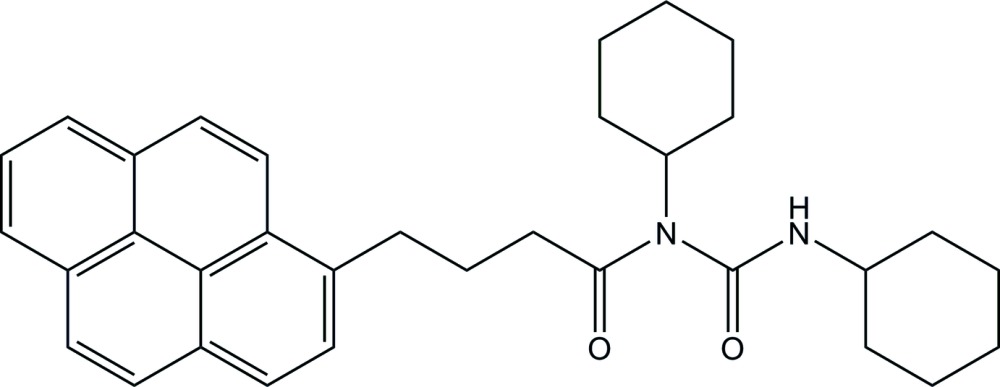


## Experimental   

### Crystal data  

C_33_H_38_N_2_O_2_
*M* *_r_* = 494.65
Triclinic, 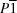

*a* = 9.0505 (15) Å
*b* = 10.1845 (17) Å
*c* = 14.571 (2) Å
α = 99.541 (3)°
β = 90.315 (3)°
γ = 92.191 (3)°
*V* = 1323.4 (4) Å^3^
*Z* = 2
Mo *K*α radiation
μ = 0.08 mm^−1^
*T* = 100 K
0.16 × 0.13 × 0.11 mm

### Data collection  

Bruker SMART APEX CCD area-detector diffractometer
Absorption correction: multi-scan (*SADABS*; Bruker, 2000 ▸) *T*_min_ = 0.988, *T*_max_ = 0.992
12906 measured reflections
4658 independent reflections
3738 reflections with *I* > 2σ(*I*)
*R*_int_ = 0.050

### Refinement  

*R*[*F* ^2^ > 2σ(*F* ^2^)] = 0.052
*wR*(*F* ^2^) = 0.128
*S* = 1.04
4658 reflections
338 parameters
1 restraint
H atoms treated by a mixture of independent and constrained refinement
Δρ_max_ = 0.19 e Å^−3^
Δρ_min_ = −0.17 e Å^−3^

### 

Data collection: *SMART* (Bruker, 2000 ▸); cell refinement: *SAINT* (Bruker, 2000 ▸); data reduction: *SAINT*; program(s) used to solve structure: *SHELXS97* (Sheldrick, 2008 ▸); program(s) used to refine structure: *SHELXL97* (Sheldrick, 2008 ▸); molecular graphics: *SHELXTL* (Sheldrick, 2008 ▸); software used to prepare material for publication: *SHELXTL*, *DIAMOND* (Brandenburg, 1997 ▸), *PLATON* (Spek, 2009 ▸) and *publCIF* (Westrip, 2010 ▸).

## Supplementary Material

Crystal structure: contains datablock(s) I, global. DOI: 10.1107/S2056989015015996/is5410sup1.cif

Structure factors: contains datablock(s) I. DOI: 10.1107/S2056989015015996/is5410Isup2.hkl

Supporting information file. DOI: 10.1107/S2056989015015996/is5410Isup3.cml

**Fig. 1.**
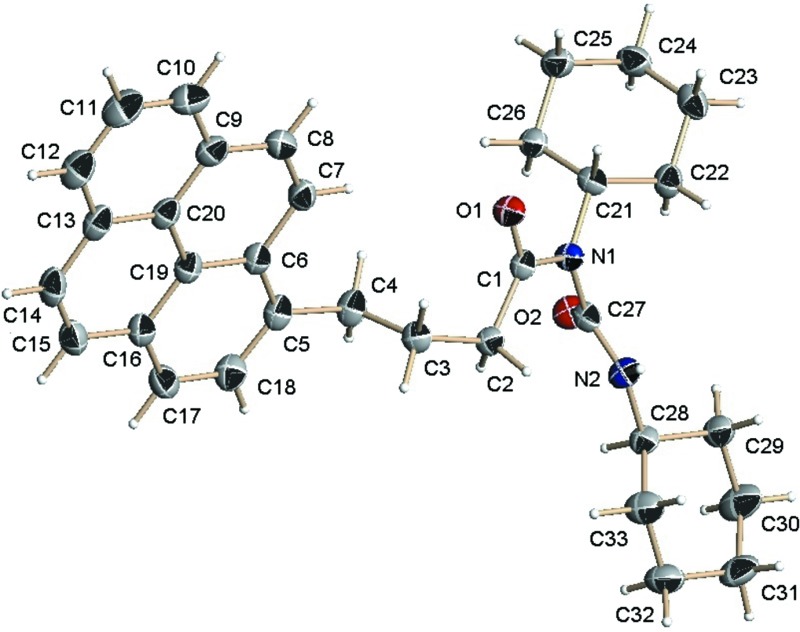
The molecular structure of the title compound, showing the atom labelling. Displacement ellipsoids are drawn at the 50% probability level.

. DOI: 10.1107/S2056989015015996/is5410fig1.tif
The mol­ecular structure of the title compound, showing the atom labelling. Displacement ellipsoids are drawn at the 50% probability level.

**Fig. 2.**
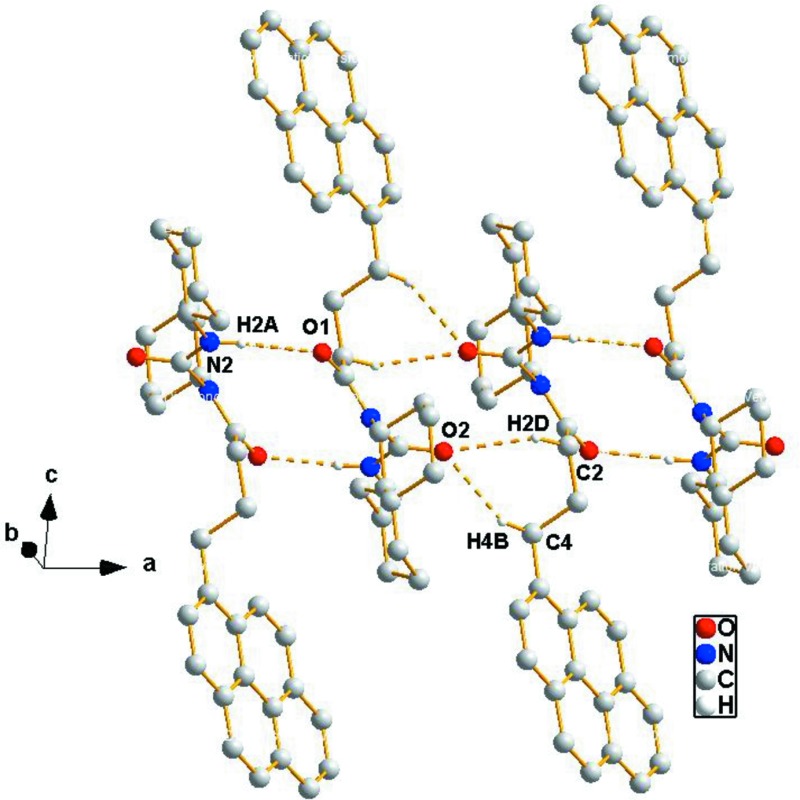
A view of the crystal packing of the title compound. Hydrogen atoms not involved in the hydrogen bonds (dashed lines) have been omitted for clarity.

. DOI: 10.1107/S2056989015015996/is5410fig2.tif
A view of the crystal packing of the title compound. Hydrogen atoms not involved in the hydrogen bonds (dashed lines) have been omitted for clarity.

CCDC reference: 1420776

Additional supporting information:  crystallographic information; 3D view; checkCIF report

## Figures and Tables

**Table 1:** Hydrogen-bond geometry (, )

*D*H*A*	*D*H	H*A*	*D* *A*	*D*H*A*
N2H2*A*O1^i^	0.86(1)	2.17(1)	3.026(2)	176(2)
C2H2*D*O2^ii^	0.99	2.49	3.358(2)	146
C4H4*B*O2^ii^	0.99	2.45	3.302(2)	144

Symmetry codes: (i) 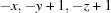; (ii) 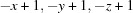.

## supplementary crystallographic information

### S1. Comment

*N*,*N*'-Dicyclohexylcarbodiimide has been used to form esters from carboxylic acid, alcohols and catalytic amounts of 2,6-dimethylpyridine (Zhu *et al.*, 2008; Gonçalves & Balogh, 2006). Nonetheless, the absence of alcohols produce the formation of *N*-acyl-*N*,*N*'-dicyclohexylureas (Kaiser *et al.*, 2008). When arenecarboxylic acids are used, the yield reaction can be modulated by electronic effects of the substituents (Slebioda, 1995). Several crystal structures of *N*-(arenecarbonyl)-*N*,*N*'-dicyclohexylurea derivatives have been reported (Chérioux *et al.*, 2002; Cai *et al.*, 2009; Imhof 2007; Dhinaa *et al.*, 2010; Pinheiro *et al.*, 2011). Herein, we now report the crystal structure of 1,3-dicyclohexyl-1-[(1-pyrenepropyl)carbonyl]urea (I).

In the molecular structure of I, the pyrenyl group and the two planes involving urea nitrogen atoms N1 and N2, C27/N1/C21/C1 and C28/N2/C27/H2A, are almost planar with r.m.s. deviations of 0.008 (2), 0.0346 (18) and 0.0098 (18) Å, respectively. The interplanar angle between the C27/N1/C21/C1 and C28/N2/C27/H2A planes is 61.1 (6)°. Each of the cyclohexyl rings adopts a chair conformation (Fig. 1). In the crystal, two neighboring molecules are linked by a pair of N—H···O interactions, generating an inversion dimer. The dimers are interconnected by C—H···O hydrogen bonds into a supramolecular chain along the *a* axis (Fig. 2 and Table 1).

### S2. Experimental

Compound I was obtained according to the literature (Abd-El-Aziz *et al.*, 2013) from an incomplete esterification reaction between pyrenobutanoic acid (6.80 mmol), *N*,*N*'-dicyclohexylcarbodiimide (7.48 mmol), 2,6-dimethylpyridine (1.08 mmol) as catalyst and 2-(thiophene-3-yl) ethanol (13.6 mmol). The three first components were stirred under room temperature for 1.5 h using 40 ml of toluene, then the last component was added and heated 1 h under reflux. (I) was isolated in a yield *ca* 8% from a column chromatography using hexane-ethyl acetate system 4:1. From slow evaporation of the mixture solution, suitable crystals for X-ray diffraction were obtained (*m.p.* = 164 °C).

### S3. Refinement

H atoms were positioned geometrically [C—H = 0.95 Å (aryl), 0.99 Å (methylene) and 1.00 Å (methine)] and constrained using a riding-model approximation with *U*_iso_(H) = 1.2*U*_eq_(C). The H atom bonded to N (H2A) was located in a difference Fourier map and refined freely with an N—H distance restraint of 0.86 (1) Å.

### Crystal data

**Table:**

C_33_H_38_N_2_O_2_	*Z* = 2
*M**_r_* = 494.65	*F*(000) = 532
Triclinic, *P*1	*D*_x_ = 1.241 Mg m^−^^3^
Hall symbol: -P 1	Mo *K*α radiation, λ = 0.71073 Å
*a* = 9.0505 (15) Å	Cell parameters from 5456 reflections
*b* = 10.1845 (17) Å	θ = 2.6–28.2°
*c* = 14.571 (2) Å	µ = 0.08 mm^−^^1^
α = 99.541 (3)°	*T* = 100 K
β = 90.315 (3)°	Plates, colourless
γ = 92.191 (3)°	0.16 × 0.13 × 0.11 mm
*V* = 1323.4 (4) Å^3^	

### Data collection

**Table:**

Bruker SMART APEX CCD area-detector diffractometer	4658 independent reflections
Radiation source: fine-focus sealed tube	3738 reflections with *I* > 2σ(*I*)
Graphite monochromator	*R*_int_ = 0.050
Detector resolution: 8.3 pixels mm^-1^	θ_max_ = 25.0°, θ_min_ = 1.4°
phi and ω scans	*h* = −10→10
Absorption correction: multi-scan (*SADABS*; Bruker, 2000)	*k* = −12→12
*T*_min_ = 0.988, *T*_max_ = 0.992	*l* = −17→17
12906 measured reflections	

### Refinement

**Table:**

Refinement on *F*^2^	Primary atom site location: structure-invariant direct methods
Least-squares matrix: full	Secondary atom site location: difference Fourier map
*R*[*F*^2^ > 2σ(*F*^2^)] = 0.052	Hydrogen site location: inferred from neighbouring sites
*wR*(*F*^2^) = 0.128	H atoms treated by a mixture of independent and constrained refinement
*S* = 1.04	*w* = 1/[σ^2^(*F*_o_^2^) + (0.031*P*)^2^ + 0.3784*P*] where *P* = (*F*_o_^2^ + 2*F*_c_^2^)/3
4658 reflections	(Δ/σ)_max_ = 0.001
338 parameters	Δρ_max_ = 0.19 e Å^−^^3^
1 restraint	Δρ_min_ = −0.17 e Å^−^^3^

### Special details

**Table:**

Experimental. ^1^H NMR (400 MHz, CDCl_3_) δ: 8.29 (d, *J* = 9.2 Hz, 2H), 8.15 (d, *J* = 7.8 Hz, 2H), 8.10 (dd, *J* = 7.7, 6.8 Hz, 2H), 8.0 (t, *J*= 7.7 Hz, 1H), 7.85 (d, *J* = 7.8 HZ, 2H), 5.27 (s, 1H), 3.90 (qn, J = 7.0 Hz, 2H), 3.36 (t, J =7.2 Hz, 2H), 2.38 (t, J = 7.2 Hz, 2H), 2.18 (qn, J = 7.2 Hz, 2H), 0.6–1.8 (m, 20H). IR (KBr) (cm^-1^) = 3299 (*w*), 2930 (*m*), 2859 (*w*), 1702 (*s*), 1633 (*s*), 1534 (*m*), 1365 (*m*), 1239(*m*), 835 (*s*). EI—MS m/*z* (%): 494 (*M*+, 5), 369 (50), 228 (100), 215 (45).
Geometry. All e.s.d.'s (except the e.s.d. in the dihedral angle between two l.s. planes) are estimated using the full covariance matrix. The cell e.s.d.'s are taken into account individually in the estimation of e.s.d.'s in distances, angles and torsion angles; correlations between e.s.d.'s in cell parameters are only used when they are defined by crystal symmetry. An approximate (isotropic) treatment of cell e.s.d.'s is used for estimating e.s.d.'s involving l.s. planes.
Refinement. Refinement of *F*^2^ against ALL reflections. The weighted *R*-factor *wR* and goodness of fit *S* are based on *F*^2^, conventional *R*-factors *R* are based on *F*, with *F* set to zero for negative *F*^2^. The threshold expression of *F*^2^ > σ(*F*^2^) is used only for calculating *R*-factors(gt) *etc*. and is not relevant to the choice of reflections for refinement. *R*-factors based on *F*^2^ are statistically about twice as large as those based on *F*, and *R*-factors based on ALL data will be even larger.

### Fractional atomic coordinates and isotropic or equivalent isotropic displacement parameters (Å^2^)

**Table:**

	*x*	*y*	*z*	*U*_iso_*/*U*_eq_	
C1	0.15199 (19)	0.48523 (17)	0.56198 (11)	0.0247 (4)	
C2	0.1888 (2)	0.63158 (17)	0.56245 (12)	0.0265 (4)	
H2C	0.1232	0.6644	0.5173	0.032*	
H2D	0.2920	0.6421	0.5418	0.032*	
C3	0.1715 (2)	0.71654 (18)	0.65839 (12)	0.0295 (4)	
H3A	0.1728	0.8117	0.6516	0.035*	
H3B	0.0743	0.6941	0.6839	0.035*	
C4	0.2932 (2)	0.6959 (2)	0.72692 (12)	0.0351 (5)	
H4A	0.2843	0.6033	0.7394	0.042*	
H4B	0.3906	0.7079	0.6983	0.042*	
C5	0.2863 (2)	0.79106 (19)	0.81785 (12)	0.0322 (4)	
C6	0.1854 (2)	0.76932 (18)	0.88786 (12)	0.0292 (4)	
C7	0.0873 (2)	0.65401 (18)	0.87947 (13)	0.0336 (5)	
H7	0.0897	0.5889	0.8247	0.040*	
C8	−0.0083 (2)	0.63533 (19)	0.94717 (13)	0.0366 (5)	
H8	−0.0714	0.5575	0.9387	0.044*	
C9	−0.0176 (2)	0.72924 (19)	1.03139 (13)	0.0348 (5)	
C10	−0.1161 (2)	0.7118 (2)	1.10233 (15)	0.0450 (5)	
H10	−0.1796	0.6342	1.0956	0.054*	
C11	−0.1221 (3)	0.8056 (2)	1.18188 (15)	0.0508 (6)	
H11	−0.1890	0.7918	1.2297	0.061*	
C12	−0.0319 (2)	0.9196 (2)	1.19279 (14)	0.0440 (5)	
H12	−0.0386	0.9838	1.2478	0.053*	
C13	0.0690 (2)	0.94226 (19)	1.12456 (12)	0.0346 (5)	
C14	0.1642 (2)	1.0593 (2)	1.13308 (13)	0.0392 (5)	
H14	0.1596	1.1249	1.1875	0.047*	
C15	0.2598 (2)	1.0785 (2)	1.06597 (14)	0.0381 (5)	
H15	0.3211	1.1574	1.0739	0.046*	
C16	0.2715 (2)	0.98252 (18)	0.98232 (13)	0.0319 (4)	
C17	0.3699 (2)	1.0003 (2)	0.91197 (13)	0.0382 (5)	
H17	0.4333	1.0780	0.9188	0.046*	
C18	0.3764 (2)	0.9061 (2)	0.83215 (13)	0.0383 (5)	
H18	0.4449	0.9207	0.7854	0.046*	
C19	0.1783 (2)	0.86558 (18)	0.97074 (12)	0.0296 (4)	
C20	0.0770 (2)	0.84527 (18)	1.04232 (12)	0.0301 (4)	
C21	0.20051 (19)	0.25014 (17)	0.50327 (12)	0.0272 (4)	
H21	0.1011	0.2347	0.5300	0.033*	
C22	0.2051 (2)	0.16123 (18)	0.40814 (12)	0.0329 (5)	
H22A	0.1279	0.1870	0.3669	0.040*	
H22B	0.3024	0.1735	0.3794	0.040*	
C23	0.1795 (2)	0.01573 (19)	0.41794 (14)	0.0376 (5)	
H23A	0.0779	0.0019	0.4402	0.045*	
H23B	0.1890	−0.0409	0.3563	0.045*	
C24	0.2896 (2)	−0.02592 (19)	0.48565 (14)	0.0396 (5)	
H24A	0.3900	−0.0238	0.4592	0.048*	
H24B	0.2648	−0.1186	0.4943	0.048*	
C25	0.2887 (2)	0.06563 (18)	0.57992 (13)	0.0374 (5)	
H25A	0.1922	0.0548	0.6100	0.045*	
H25B	0.3668	0.0398	0.6206	0.045*	
C26	0.3151 (2)	0.21121 (18)	0.56959 (12)	0.0305 (4)	
H26A	0.3083	0.2686	0.6312	0.037*	
H26B	0.4156	0.2244	0.5452	0.037*	
C27	0.3174 (2)	0.42850 (17)	0.42681 (12)	0.0264 (4)	
C28	0.3480 (2)	0.54841 (18)	0.29600 (12)	0.0290 (4)	
H28	0.4519	0.5645	0.3200	0.035*	
C29	0.3498 (2)	0.44741 (19)	0.20684 (12)	0.0368 (5)	
H29A	0.2471	0.4248	0.1844	0.044*	
H29B	0.3940	0.3648	0.2197	0.044*	
C30	0.4382 (3)	0.5017 (2)	0.13153 (15)	0.0547 (6)	
H30A	0.5438	0.5123	0.1505	0.066*	
H30B	0.4303	0.4367	0.0729	0.066*	
C31	0.3839 (3)	0.6349 (2)	0.11436 (14)	0.0485 (6)	
H31A	0.4491	0.6700	0.0690	0.058*	
H31B	0.2828	0.6221	0.0872	0.058*	
C32	0.3822 (3)	0.7348 (2)	0.20321 (14)	0.0459 (5)	
H32A	0.3393	0.8181	0.1906	0.055*	
H32B	0.4848	0.7562	0.2263	0.055*	
C33	0.2913 (3)	0.6800 (2)	0.27775 (14)	0.0450 (6)	
H33A	0.2962	0.7454	0.3362	0.054*	
H33B	0.1866	0.6673	0.2572	0.054*	
H2A	0.1664 (11)	0.5120 (18)	0.3687 (12)	0.028 (5)*	
N1	0.21491 (16)	0.39366 (14)	0.49557 (10)	0.0257 (3)	
N2	0.25912 (17)	0.49893 (15)	0.36770 (10)	0.0297 (4)	
O1	0.06756 (13)	0.44841 (12)	0.61964 (8)	0.0314 (3)	
O2	0.44266 (14)	0.38979 (13)	0.42418 (9)	0.0353 (3)	

### Atomic displacement parameters (Å^2^)

**Table:**

	*U*^11^	*U*^22^	*U*^33^	*U*^12^	*U*^13^	*U*^23^
C1	0.0230 (9)	0.0294 (10)	0.0212 (9)	0.0054 (7)	0.0028 (7)	0.0014 (8)
C2	0.0285 (10)	0.0267 (10)	0.0242 (9)	0.0052 (8)	0.0049 (7)	0.0023 (7)
C3	0.0317 (10)	0.0272 (10)	0.0286 (10)	0.0072 (8)	0.0063 (8)	−0.0004 (8)
C4	0.0336 (11)	0.0422 (12)	0.0277 (10)	0.0115 (9)	0.0044 (8)	−0.0015 (9)
C5	0.0297 (10)	0.0386 (11)	0.0272 (10)	0.0099 (9)	0.0001 (8)	0.0002 (8)
C6	0.0317 (10)	0.0283 (10)	0.0269 (10)	0.0085 (8)	−0.0017 (8)	0.0013 (8)
C7	0.0445 (12)	0.0273 (10)	0.0279 (10)	0.0052 (9)	−0.0016 (9)	0.0004 (8)
C8	0.0429 (12)	0.0308 (11)	0.0366 (11)	−0.0016 (9)	−0.0027 (9)	0.0076 (9)
C9	0.0381 (11)	0.0363 (11)	0.0319 (11)	0.0075 (9)	0.0010 (9)	0.0098 (9)
C10	0.0472 (13)	0.0451 (13)	0.0462 (13)	0.0027 (10)	0.0095 (10)	0.0167 (11)
C11	0.0553 (15)	0.0647 (16)	0.0355 (12)	0.0115 (12)	0.0179 (11)	0.0143 (11)
C12	0.0501 (14)	0.0526 (14)	0.0292 (11)	0.0161 (11)	0.0081 (10)	0.0029 (10)
C13	0.0403 (12)	0.0391 (12)	0.0247 (10)	0.0163 (9)	−0.0002 (8)	0.0027 (8)
C14	0.0487 (13)	0.0371 (12)	0.0285 (11)	0.0123 (10)	−0.0054 (9)	−0.0064 (9)
C15	0.0436 (12)	0.0320 (11)	0.0362 (11)	0.0028 (9)	−0.0073 (9)	−0.0017 (9)
C16	0.0314 (11)	0.0331 (11)	0.0301 (10)	0.0037 (8)	−0.0035 (8)	0.0014 (8)
C17	0.0362 (11)	0.0383 (12)	0.0385 (11)	−0.0063 (9)	−0.0028 (9)	0.0031 (9)
C18	0.0318 (11)	0.0506 (13)	0.0314 (11)	0.0012 (9)	0.0052 (9)	0.0036 (9)
C19	0.0308 (10)	0.0320 (10)	0.0261 (10)	0.0088 (8)	−0.0019 (8)	0.0032 (8)
C20	0.0335 (11)	0.0332 (11)	0.0247 (10)	0.0108 (8)	0.0000 (8)	0.0054 (8)
C21	0.0272 (10)	0.0246 (9)	0.0288 (10)	0.0024 (7)	0.0088 (8)	0.0010 (8)
C22	0.0364 (11)	0.0321 (11)	0.0288 (10)	0.0077 (8)	−0.0003 (8)	−0.0010 (8)
C23	0.0399 (12)	0.0310 (11)	0.0378 (11)	0.0002 (9)	0.0037 (9)	−0.0061 (9)
C24	0.0507 (13)	0.0260 (10)	0.0418 (12)	0.0043 (9)	0.0041 (10)	0.0038 (9)
C25	0.0484 (13)	0.0303 (11)	0.0342 (11)	0.0031 (9)	0.0027 (9)	0.0071 (9)
C26	0.0361 (11)	0.0294 (10)	0.0254 (10)	0.0012 (8)	0.0039 (8)	0.0024 (8)
C27	0.0291 (10)	0.0242 (9)	0.0237 (9)	0.0036 (8)	0.0084 (8)	−0.0034 (7)
C28	0.0310 (10)	0.0320 (10)	0.0238 (9)	0.0017 (8)	0.0088 (8)	0.0039 (8)
C29	0.0515 (13)	0.0306 (11)	0.0277 (10)	0.0058 (9)	0.0065 (9)	0.0018 (8)
C30	0.0814 (18)	0.0509 (14)	0.0338 (12)	0.0183 (13)	0.0249 (12)	0.0081 (10)
C31	0.0625 (15)	0.0533 (14)	0.0340 (12)	0.0037 (11)	0.0122 (10)	0.0188 (10)
C32	0.0595 (15)	0.0352 (12)	0.0458 (13)	0.0034 (10)	0.0098 (11)	0.0140 (10)
C33	0.0628 (15)	0.0336 (12)	0.0392 (12)	0.0114 (10)	0.0191 (11)	0.0050 (9)
N1	0.0285 (8)	0.0238 (8)	0.0245 (8)	0.0048 (6)	0.0091 (6)	0.0018 (6)
N2	0.0272 (9)	0.0360 (9)	0.0268 (8)	0.0081 (7)	0.0095 (7)	0.0061 (7)
O1	0.0335 (7)	0.0323 (7)	0.0280 (7)	0.0041 (6)	0.0136 (6)	0.0023 (6)
O2	0.0284 (7)	0.0413 (8)	0.0371 (8)	0.0081 (6)	0.0103 (6)	0.0075 (6)

### Geometric parameters (Å, º)

**Table:**

C1—O1	1.2329 (19)	C21—N1	1.485 (2)
C1—N1	1.370 (2)	C21—C26	1.521 (2)
C1—C2	1.514 (2)	C21—C22	1.527 (2)
C2—C3	1.529 (2)	C21—H21	1.0000
C2—H2C	0.9900	C22—C23	1.521 (3)
C2—H2D	0.9900	C22—H22A	0.9900
C3—C4	1.526 (3)	C22—H22B	0.9900
C3—H3A	0.9900	C23—C24	1.518 (3)
C3—H3B	0.9900	C23—H23A	0.9900
C4—C5	1.510 (2)	C23—H23B	0.9900
C4—H4A	0.9900	C24—C25	1.527 (3)
C4—H4B	0.9900	C24—H24A	0.9900
C5—C18	1.388 (3)	C24—H24B	0.9900
C5—C6	1.412 (2)	C25—C26	1.525 (3)
C6—C19	1.427 (2)	C25—H25A	0.9900
C6—C7	1.433 (3)	C25—H25B	0.9900
C7—C8	1.348 (3)	C26—H26A	0.9900
C7—H7	0.9500	C26—H26B	0.9900
C8—C9	1.430 (3)	C27—O2	1.213 (2)
C8—H8	0.9500	C27—N2	1.328 (2)
C9—C10	1.398 (3)	C27—N1	1.447 (2)
C9—C20	1.419 (3)	C28—N2	1.464 (2)
C10—C11	1.378 (3)	C28—C33	1.516 (3)
C10—H10	0.9500	C28—C29	1.518 (2)
C11—C12	1.380 (3)	C28—H28	1.0000
C11—H11	0.9500	C29—C30	1.525 (3)
C12—C13	1.394 (3)	C29—H29A	0.9900
C12—H12	0.9500	C29—H29B	0.9900
C13—C20	1.425 (2)	C30—C31	1.520 (3)
C13—C14	1.432 (3)	C30—H30A	0.9900
C14—C15	1.343 (3)	C30—H30B	0.9900
C14—H14	0.9500	C31—C32	1.509 (3)
C15—C16	1.438 (3)	C31—H31A	0.9900
C15—H15	0.9500	C31—H31B	0.9900
C16—C17	1.391 (3)	C32—C33	1.531 (3)
C16—C19	1.419 (3)	C32—H32A	0.9900
C17—C18	1.382 (3)	C32—H32B	0.9900
C17—H17	0.9500	C33—H33A	0.9900
C18—H18	0.9500	C33—H33B	0.9900
C19—C20	1.428 (2)	N2—H2A	0.855 (9)
			
O1—C1—N1	120.38 (16)	C23—C22—C21	110.36 (15)
O1—C1—C2	121.25 (14)	C23—C22—H22A	109.6
N1—C1—C2	118.37 (14)	C21—C22—H22A	109.6
C1—C2—C3	112.77 (14)	C23—C22—H22B	109.6
C1—C2—H2C	109.0	C21—C22—H22B	109.6
C3—C2—H2C	109.0	H22A—C22—H22B	108.1
C1—C2—H2D	109.0	C24—C23—C22	111.45 (16)
C3—C2—H2D	109.0	C24—C23—H23A	109.3
H2C—C2—H2D	107.8	C22—C23—H23A	109.3
C4—C3—C2	112.71 (14)	C24—C23—H23B	109.3
C4—C3—H3A	109.0	C22—C23—H23B	109.3
C2—C3—H3A	109.0	H23A—C23—H23B	108.0
C4—C3—H3B	109.0	C23—C24—C25	111.59 (16)
C2—C3—H3B	109.0	C23—C24—H24A	109.3
H3A—C3—H3B	107.8	C25—C24—H24A	109.3
C5—C4—C3	112.66 (15)	C23—C24—H24B	109.3
C5—C4—H4A	109.1	C25—C24—H24B	109.3
C3—C4—H4A	109.1	H24A—C24—H24B	108.0
C5—C4—H4B	109.1	C26—C25—C24	111.40 (15)
C3—C4—H4B	109.1	C26—C25—H25A	109.3
H4A—C4—H4B	107.8	C24—C25—H25A	109.3
C18—C5—C6	118.59 (16)	C26—C25—H25B	109.3
C18—C5—C4	119.68 (17)	C24—C25—H25B	109.3
C6—C5—C4	121.66 (17)	H25A—C25—H25B	108.0
C5—C6—C19	119.51 (17)	C21—C26—C25	109.86 (15)
C5—C6—C7	122.80 (16)	C21—C26—H26A	109.7
C19—C6—C7	117.69 (16)	C25—C26—H26A	109.7
C8—C7—C6	121.81 (17)	C21—C26—H26B	109.7
C8—C7—H7	119.1	C25—C26—H26B	109.7
C6—C7—H7	119.1	H26A—C26—H26B	108.2
C7—C8—C9	121.97 (18)	O2—C27—N2	125.42 (16)
C7—C8—H8	119.0	O2—C27—N1	120.55 (16)
C9—C8—H8	119.0	N2—C27—N1	113.96 (15)
C10—C9—C20	119.10 (18)	N2—C28—C33	110.08 (14)
C10—C9—C8	122.84 (19)	N2—C28—C29	111.64 (15)
C20—C9—C8	118.06 (17)	C33—C28—C29	110.95 (16)
C11—C10—C9	120.8 (2)	N2—C28—H28	108.0
C11—C10—H10	119.6	C33—C28—H28	108.0
C9—C10—H10	119.6	C29—C28—H28	108.0
C10—C11—C12	120.65 (19)	C28—C29—C30	111.25 (16)
C10—C11—H11	119.7	C28—C29—H29A	109.4
C12—C11—H11	119.7	C30—C29—H29A	109.4
C11—C12—C13	121.16 (19)	C28—C29—H29B	109.4
C11—C12—H12	119.4	C30—C29—H29B	109.4
C13—C12—H12	119.4	H29A—C29—H29B	108.0
C12—C13—C20	118.67 (19)	C31—C30—C29	111.98 (17)
C12—C13—C14	122.89 (18)	C31—C30—H30A	109.2
C20—C13—C14	118.43 (17)	C29—C30—H30A	109.2
C15—C14—C13	121.50 (18)	C31—C30—H30B	109.2
C15—C14—H14	119.2	C29—C30—H30B	109.2
C13—C14—H14	119.2	H30A—C30—H30B	107.9
C14—C15—C16	121.61 (19)	C32—C31—C30	111.46 (17)
C14—C15—H15	119.2	C32—C31—H31A	109.3
C16—C15—H15	119.2	C30—C31—H31A	109.3
C17—C16—C19	118.72 (16)	C32—C31—H31B	109.3
C17—C16—C15	122.57 (18)	C30—C31—H31B	109.3
C19—C16—C15	118.71 (17)	H31A—C31—H31B	108.0
C18—C17—C16	120.66 (18)	C31—C32—C33	110.94 (17)
C18—C17—H17	119.7	C31—C32—H32A	109.5
C16—C17—H17	119.7	C33—C32—H32A	109.5
C17—C18—C5	122.37 (18)	C31—C32—H32B	109.5
C17—C18—H18	118.8	C33—C32—H32B	109.5
C5—C18—H18	118.8	H32A—C32—H32B	108.0
C16—C19—C6	120.14 (16)	C28—C33—C32	111.45 (16)
C16—C19—C20	119.56 (16)	C28—C33—H33A	109.3
C6—C19—C20	120.30 (17)	C32—C33—H33A	109.3
C9—C20—C13	119.65 (17)	C28—C33—H33B	109.3
C9—C20—C19	120.16 (16)	C32—C33—H33B	109.3
C13—C20—C19	120.18 (18)	H33A—C33—H33B	108.0
N1—C21—C26	112.10 (14)	C1—N1—C27	123.78 (14)
N1—C21—C22	111.76 (14)	C1—N1—C21	119.09 (14)
C26—C21—C22	111.18 (14)	C27—N1—C21	116.20 (13)
N1—C21—H21	107.2	C27—N2—C28	121.70 (15)
C26—C21—H21	107.2	C27—N2—H2A	119.8 (12)
C22—C21—H21	107.2	C28—N2—H2A	118.4 (12)
			
O1—C1—C2—C3	25.2 (2)	C8—C9—C20—C19	−0.3 (3)
N1—C1—C2—C3	−154.78 (15)	C12—C13—C20—C9	0.5 (3)
C1—C2—C3—C4	72.4 (2)	C14—C13—C20—C9	−179.06 (17)
C2—C3—C4—C5	173.33 (16)	C12—C13—C20—C19	179.60 (16)
C3—C4—C5—C18	−97.4 (2)	C14—C13—C20—C19	0.1 (3)
C3—C4—C5—C6	79.7 (2)	C16—C19—C20—C9	179.55 (16)
C18—C5—C6—C19	0.2 (3)	C6—C19—C20—C9	−0.2 (3)
C4—C5—C6—C19	−176.96 (16)	C16—C19—C20—C13	0.4 (3)
C18—C5—C6—C7	179.69 (17)	C6—C19—C20—C13	−179.31 (16)
C4—C5—C6—C7	2.5 (3)	N1—C21—C22—C23	−176.13 (15)
C5—C6—C7—C8	−179.91 (17)	C26—C21—C22—C23	57.8 (2)
C19—C6—C7—C8	−0.4 (3)	C21—C22—C23—C24	−55.6 (2)
C6—C7—C8—C9	0.0 (3)	C22—C23—C24—C25	54.4 (2)
C7—C8—C9—C10	179.94 (18)	C23—C24—C25—C26	−54.8 (2)
C7—C8—C9—C20	0.4 (3)	N1—C21—C26—C25	176.17 (14)
C20—C9—C10—C11	0.2 (3)	C22—C21—C26—C25	−57.92 (19)
C8—C9—C10—C11	−179.37 (19)	C24—C25—C26—C21	56.1 (2)
C9—C10—C11—C12	0.6 (3)	N2—C28—C29—C30	178.07 (16)
C10—C11—C12—C13	−0.9 (3)	C33—C28—C29—C30	54.9 (2)
C11—C12—C13—C20	0.3 (3)	C28—C29—C30—C31	−54.2 (3)
C11—C12—C13—C14	179.83 (19)	C29—C30—C31—C32	54.4 (3)
C12—C13—C14—C15	−179.73 (19)	C30—C31—C32—C33	−54.9 (3)
C20—C13—C14—C15	−0.2 (3)	N2—C28—C33—C32	179.84 (17)
C13—C14—C15—C16	−0.1 (3)	C29—C28—C33—C32	−56.1 (2)
C14—C15—C16—C17	−179.91 (18)	C31—C32—C33—C28	56.2 (2)
C14—C15—C16—C19	0.6 (3)	O1—C1—N1—C27	−178.60 (15)
C19—C16—C17—C18	0.2 (3)	C2—C1—N1—C27	1.4 (2)
C15—C16—C17—C18	−179.24 (18)	O1—C1—N1—C21	−10.1 (2)
C16—C17—C18—C5	0.3 (3)	C2—C1—N1—C21	169.94 (15)
C6—C5—C18—C17	−0.5 (3)	O2—C27—N1—C1	118.43 (19)
C4—C5—C18—C17	176.71 (18)	N2—C27—N1—C1	−64.3 (2)
C17—C16—C19—C6	−0.5 (3)	O2—C27—N1—C21	−50.4 (2)
C15—C16—C19—C6	178.97 (16)	N2—C27—N1—C21	126.88 (16)
C17—C16—C19—C20	179.76 (17)	C26—C21—N1—C1	−82.44 (19)
C15—C16—C19—C20	−0.7 (3)	C22—C21—N1—C1	151.97 (15)
C5—C6—C19—C16	0.3 (3)	C26—C21—N1—C27	86.94 (18)
C7—C6—C19—C16	−179.21 (16)	C22—C21—N1—C27	−38.7 (2)
C5—C6—C19—C20	−179.97 (16)	O2—C27—N2—C28	−5.7 (3)
C7—C6—C19—C20	0.5 (2)	N1—C27—N2—C28	177.15 (14)
C10—C9—C20—C13	−0.7 (3)	C33—C28—N2—C27	−147.59 (18)
C8—C9—C20—C13	178.86 (17)	C29—C28—N2—C27	88.7 (2)
C10—C9—C20—C19	−179.85 (17)		

### Hydrogen-bond geometry (Å, º)

**Table:**

*D*—H···*A*	*D*—H	H···*A*	*D*···*A*	*D*—H···*A*
N2—H2*A*···O1^i^	0.86 (1)	2.17 (1)	3.026 (2)	176 (2)
C2—H2*D*···O2^ii^	0.99	2.49	3.358 (2)	146
C4—H4*B*···O2^ii^	0.99	2.45	3.302 (2)	144

Symmetry codes: (i) −*x*, −*y*+1, −*z*+1; (ii) −*x*+1, −*y*+1, −*z*+1.
