# Supplementary figures and images for: Crystal structure of 1,3-di­cyclo­hexyl-1-[3-(pyren-1-yl)prop­anoyl]urea
Source: Acta Crystallogr E Crystallogr Commun. 2015 Sep 12;71(Pt 10):o737–8. doi: 10.1107/S2056989015015996 (PMC4647353; doi:10.1107/S2056989015015996)

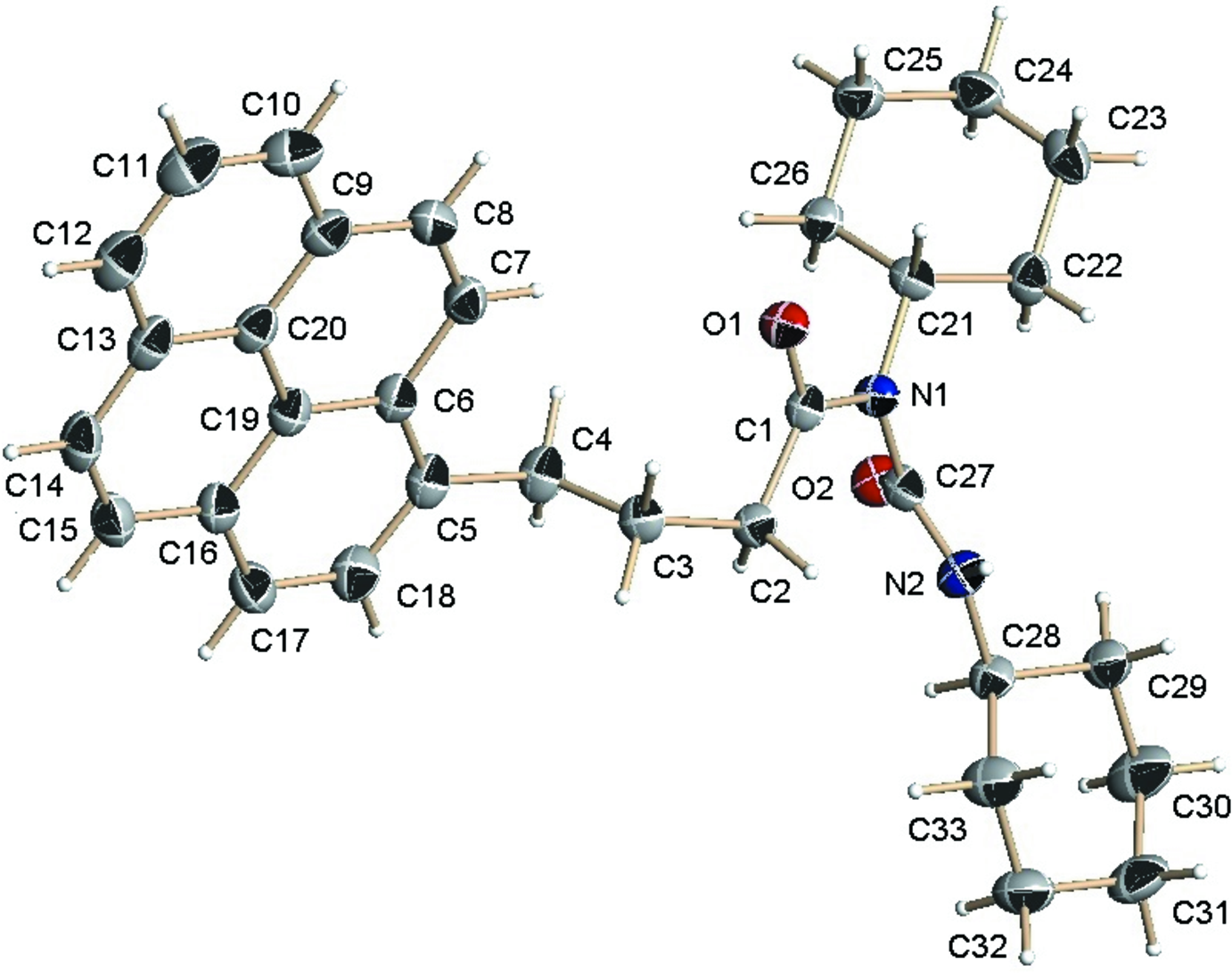

Supplement: Supplementary file 4 [file e-71-0o737-fig1.tif]

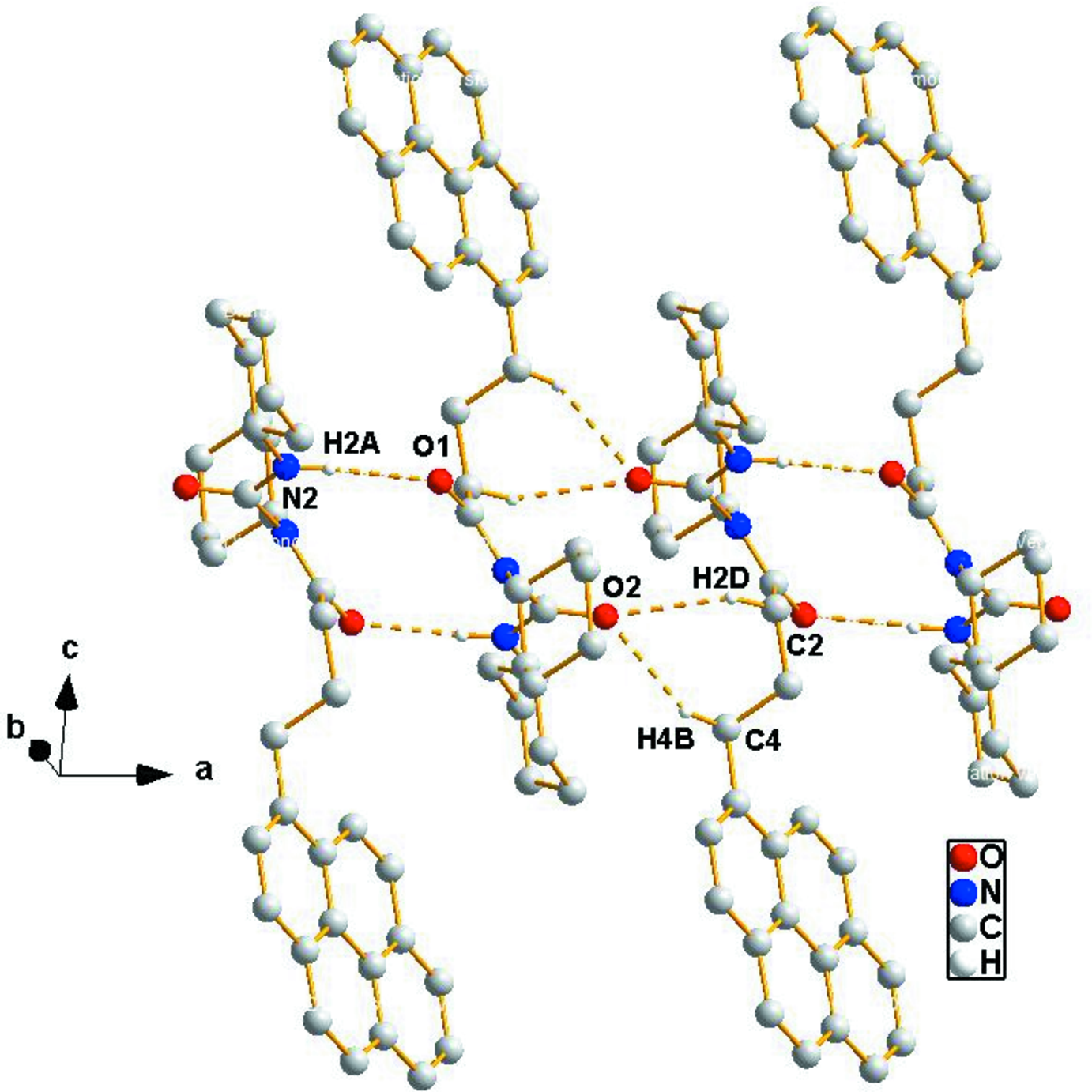

Supplement: Supplementary file 5 [file e-71-0o737-fig2.tif]
